# An Equity-Driven Analysis of the Implications of Attachment and Attachment Readiness Within Primary Care Healthcare Systems in Canada

**DOI:** 10.3390/healthcare14172840

**Published:** 2026-09-03

**Authors:** Akm Alamgir, Subrana Rahman, Saleema Allana, Rosanra Yoon, Kasia Filaber, Cliff Ledwos, Pema Yanzom, Faye Goldman, Michelle Naimer, Jennifer Rayner

**Affiliations:** 1 Access Alliance Multicultural Health and Community Services, Toronto, ON M5T 3A9, Canadapempemzom@gmail.com (P.Y.); 2 Arthur Labatt Family School of Nursing, Western University, London, ON N6A 3K7, Canada; sallana5@uwo.ca; 3 Lawrence Blomberg Faculty of Nursing, University of Toronto, Toronto, ON M5T 1P8, Canada; rosanra.yoon@utoronto.ca; 4 Department of Family and Community Medicine, Temerty Faculty of Medicine, University of Toronto, Toronto, ON M5T 1P8, Canada; faye.goldman@utoronto.ca (F.G.); michelle.naimer@sinaihealth.ca (M.N.); 5 Alliance for Healthier Communities, Toronto, ON M6A 3B6, Canada; jennifer.rayner@allianceon.org

**Keywords:** attachment to primary care, attachment readiness, equity in attachment, primary healthcare system, immigrants and newcomers

## Abstract

**Background**: The persistent challenge of not having a physician or nurse practitioner for ongoing care for millions of residents imposes a burden on the health economy in Canada and demands a shift in focus from mere system registry to the value-based concept of attachment readiness. With a hypothesis that true attachment is a function of attachment readiness, a relational process shaped by the intersection of patients’ preparation, providers’ capacity, and systemic support, this study aims to investigate the challenges of attachment-creating barriers to meaningful healthcare relationships. **Methods**: This sequential mixed-methods study was designed to integrate a comprehensive scoping review of academic librarian-guided databases, search engines, grey literature, and snowballing, followed by 360-degree qualitative interviews with experts, service providers, and clients. Qualitative data was analyzed by thematic coding to answer the research questions. **Results**: Attachment is a function of a multitude of factors from patients and physicians’ “attachment readiness” is a multilevel, process-oriented condition shaped by patient preparedness, provider capacity, and system design more than a single episodic event or individual attribute. Facilitators such as team-based care, patient navigation, culturally responsive outreach, and structured onboarding reinforce readiness as a system-enabled condition. However, persistent system-level barriers delay attachment among motivated patients (readiness decay), leading to frustration, disengagement, and reduced trust. Findings further suggest that attachment is constrained not only by physician supply but also by time-sensitive readiness conditions. **Conclusions**: This study suggests that attachment to primary care is one leg of a three-legged stool, upheld and sustained by the two others- equity and access. To facilitate true attachment and to move beyond fragmented care and reliance on high-cost emergency departments, Canadian healthcare policy must consider attachment readiness through standardizing onboarding (implementing uniform digital intake processes to reduce repetitive data entry and administrative friction), an equity lens for prioritizing vulnerability (refining centralized waitlist algorithms to account for clinical urgency and social determinants of health), and supporting provider capacity (providing financial and administrative incentives that recognize the labour involved in establishing new longitudinal relationships for patients with complex needs).

## 1. Background

According to the Canadian Medical Association (CMA), approximately 6.5 million Canadians remain unattached to ongoing primary care, which is the essential front door to the healthcare system in Canada and foundational to accessing timely preventive care and chronic disease management [1]. This represents more than 15% of the population, indicating limited consistent access to primary care [2]. Certain groups, such as immigrants, low-income individuals, formerly incarcerated people, people with a history of opioid use, and those experiencing mental health challenges, are disproportionately less likely to have a regular primary care provider [3,4,5]. Patients who are not attached to a primary care provider often rely on walk-in clinics or emergency departments for both basic and preventive health services [6,7].

Canada ranks among the lowest in primary care attachment rates within the Organization for Economic Co-operation and Development (OECD) countries [8]. This lack of attachment to primary care providers limits access to healthcare and diminishes the quality of care, largely due to disruption in system navigation and fragmented care [9]. For residents without a consistent healthcare provider, it is a significant contributor to preventable health problems [9]. Those without a regular family doctor or nurse practitioner face substantial barriers to care. Research by Crooks, Agarwal, and Harrison [10] revealed that unattached individuals experience fragmented medical records, limited access to specialized care due to missing referrals, and the absence of a continuous doctor–patient relationship that fosters trust and communication [11]. Experiencing longer periods without attachment is associated with poorer health outcomes and higher healthcare costs, particularly among individuals with moderate-to-high comorbidities [9].

In Ontario, systemic issues, such as an ongoing severe shortage of family physicians and family health teams, compound these challenges. The Canadian Health Workforce Education, Training, and Distribution Study [12] reported a deficit of about 23,000 family doctors, with only 1700 new graduates entering the workforce each year, far below replacement levels. This shortage, along with underutilized internal training programs in Canada and an aging physician population, exacerbates the attachment gap. Primary care providers face increasing administrative burdens, outdated billing and remuneration models, and significant workforce challenges that impact both recruitment and retention. These structural challenges contribute to burnout and reduced capacity, thereby further straining access to primary care. Despite the need for more medical professionals, Ontario experiences significant brain waste, with many internationally educated professionals not hired and their skills going to waste [13]. This shortage of health professionals exacerbates the gap in healthcare access. Solo family medicine practices are overstretched and do not have the necessary resources and access to team-based care to manage the needs of complex patients experiencing multiple social, health, and structural vulnerabilities such as food insecurity, poverty, and complex co-occurring mental health or substance use challenges. Subsequently, solo family medicine practices are forced to maintain smaller rosters and selectively accept patients due to limited capacity and resources to adequately care for patients with severe, complex chronic conditions or substance use [10].

To address these challenges, governments at all levels have introduced multiple initiatives to enhance attachment to primary care. Ontario’s Primary Care Action Plan (2025–2029), a provincial government strategy, aims to connect approximately 2 million residents by creating and expanding interprofessional primary care teams [14]. Further, Ontario’s Healthcare Connect is a centralized waitlist for unattached patients awaiting attachment. Other provinces have implemented similar policies and programs. British Columbia’s provincial attachment system uses the Health Connect Registry and Provider Panel Registry to link patients and providers. Quebec’s Guichet d’accès à la première ligne (GAP) and GAMF, Nova Scotia’s Need a Family Practice Registry, Alberta’s Primary Care Networks and AlbertaFindADoctor.ca, Prince Edward Island’s Family Care Teams, and Newfoundland and Labrador’s Patient Connect NL all aim to improve equitable, longitudinal access to primary healthcare across jurisdictions.

### Problem Statement

Despite these nationwide efforts, millions of Canadians remain unattached to a consistent primary care provider. Improving access requires more than just system capacity. The readiness of both patients and providers to engage in and sustain ongoing primary care relationships is crucial but underexplored. This study explores attachment readiness from both perspectives.

This article informs strategies that streamline and strengthen the onboarding process for primary care attachment by integrating the perspectives of patients, providers, and the healthcare system to support equitable, sustainable, and person-centred care.

Research Questions:

- What are the global concepts of “Attachment” and “Attachment Readiness” to primary care?
- What is the impact of attachment and attachment readiness (attaching prepared or not prepared patients) on the healthcare system (cost of non-attachment)?
- What are the challenges to and facilitators of attachment readiness?

Specifically, this review seeks to clarify the global concepts of attachment and attachment readiness within primary care, examine the impact of attachment and non-attachment on health system outcomes, including the costs associated with unattached care, and explore the key challenges and facilitators that influence patients’ readiness to attach to a primary care provider.

## 2. Methodology

This project employed a sequential approach [15]: a scoping review to generate foundational information, followed by interviews with patients and providers based on the information gathered during the scoping review, and, finally, validation by expert consultation. A scoping review was considered the most appropriate, given the novelty of the area of study and the scarcity of relevant articles, to capture anecdotal experiences and to define clinical attachment and differentiate it from other attachments.

### 2.1. Scoping Review

This scoping review activity, conducted in September and October of 2025, followed an enhanced modelling [16] of the Arksey and O’Malley framework (2005) [17] and included peer-reviewed literature from databases, search engines such as Google Scholar, snowball searches of the references of other literature, and grey literature sources. PubMed, MEDLINE, ProQuest, ScienceDirect, and JSTOR databases were searched, following PRISMA-ScR guidance [18]. Grey literature searches were conducted through Google and other references. Databases, search terms and Boolean Operators were reviewed and guided by an academic librarian at Seneca College in Toronto. The search strategy details are available in Appendix B.

**Figure 1:**
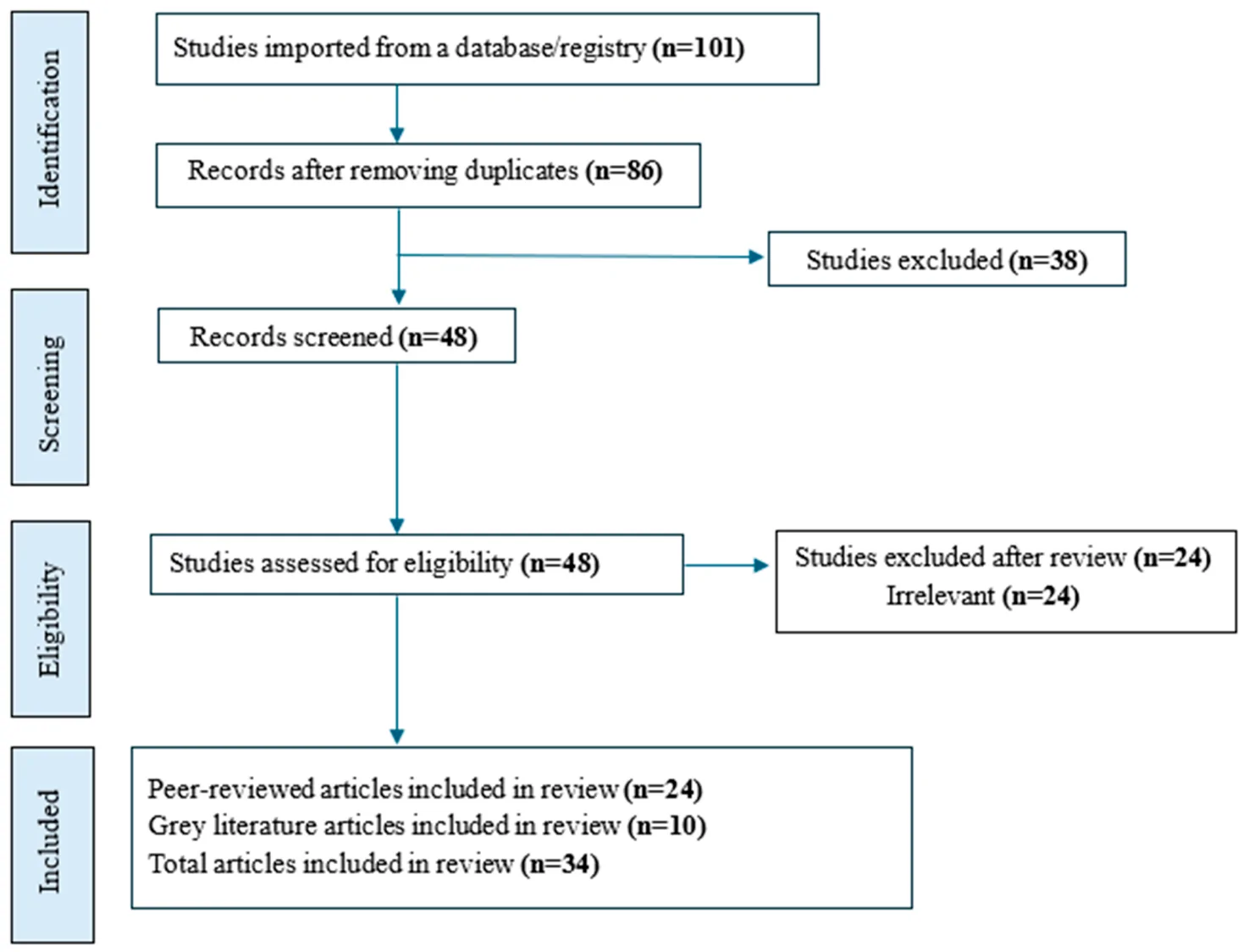
PRISMA flow diagram.

The inclusion criteria required articles in English (published in English or with an English translation available) that examined the concepts of attachment to primary care and attachment readiness within primary healthcare systems in Canada or other countries with comparable health care systems. Considering the relative scarcity of relevant literature and rapidly changing strategies for health system reforms and attachment initiatives, articles published in the last 15 years were included. Exceptions were made for seminal articles or articles of high relevance and value. Studies were excluded from this review if they did not address tools or practices that were grounded in evidence, full texts were not available, and scientific rigour was not validated during quality checks by the Critical Appraisal Skills Programme (CASP) checklist [19] (Figure 1).

The conventional five-step strategy of scoping review practice (identifying the research questions, identifying relevant studies, selecting eligible studies, charting the data, and collating, summarizing, and reporting the results) was strengthened by adding four practice-based components. First, the PRISMA-ScR guidelines were incorporated to systematically screen, sort, and select relevant articles retrieved from databases. Second, Flicker and Nixon’s collaborative data analysis approach was adopted to minimize individual researcher bias and reduce variability in interpretations among researchers. Third, the traditional role of the principal investigator was shifted to an “insider–outsider” perspective, which helped limit the investigator’s influence on the data analysis process, to resolve disputes among researchers, and to triangulate findings [20]. Finally, the CASP checklist was integrated as a quality monitoring tool to ensure the rigour and credibility of the review process and its outcomes.

Identified literature was manually screened for duplicates and for eligibility by one independent reviewer (P.Y.). Articles were then exported into MyBib (https://www.mybib.com/, accessed on 18 September 2025), a citation management website, to sort through relevant articles. Grey literature sources were assessed for relevance and credibility based on authoring organization, publication date, and alignment with the review’s focus on attachment. Specific sources include government reports and policy documents. A reflexive thematic approach was conducted on the extracted articles and included themes surrounding attachment and its impacts, facilitators, barriers, readiness, and other characteristics [21]. Reflexive thematic analysis positions researcher subjectivity as a valuable resource and views coding as inherently interpretive rather than fixed [22].

### 2.2. Qualitative—Semi-Structured Interviews

This study further employed a qualitative descriptive design using semi-structured, in-depth interviews to explore attachment readiness to primary care from the perspectives of 8 patients, two physicians from two private clinics, one physician from a community health centre and two project managers. A qualitative approach was selected to capture participants’ experiences, perceptions, and interpretations of attachment and onboarding processes within real-world primary care contexts [21].

### 2.3. Participant Sampling and Recruitment

This study used a purposive snowball sampling approach with intentional design to ensure heterogeneity and maximum variation [23]. This strategy was used to recruit participants with direct experience of primary care attachment processes. Sampling aimed to capture diverse perspectives from patients and providers relevant to attachment readiness. Sampling continued until information power was achieved, defined as the point at which additional interviews did not yield substantively new insights relevant to attachment readiness [24]. Patients were recruited through the Access Alliance Multicultural Healthcare Centre and were either randomly phoned or approached by staff or study team members, who provided information about the study. Patients were eligible to participate if they had recently sought attachment to a primary care provider or had experienced onboarding or intake processes related to primary care attachment. Providers were recruited via professional networks, organizational contacts, and email invitations. Primary healthcare providers and staff were eligible if they were involved in patient attachment, intake, onboarding, or panel management and practised in private primary care clinics, community health centres, or team-based primary care settings. Interested individuals were contacted directly by the research team.

### 2.4. Data Collection

Data collection began between October 2025 and December 2025. Interviews were conducted by a trained qualitative researcher (P.Y.). An interview guide was developed based on the study objectives and existing literature on primary care attachment and attachment readiness. The guide included open-ended questions and probes exploring perceptions of readiness, administrative processes, barriers and facilitators, and suggestions for improvement. Interviews were conducted in person or virtually (by phone/video), depending on participants’ preferences and availability. Interviews lasted approximately 30–60 min and were audio-recorded with participant consent. Field notes were taken during and immediately after interviews to capture contextual details and initial analytic impressions.

The thematic analysis was performed using an iterative, inductive approach, ensuring a reflexive process [21]. The analysis was conducted manually by one researcher (P.Y.), with codes being developed using the interview guide and discussions with the principal investigator.

## 3. Result

### 3.1. Scoping Review Findings: Characteristics of Included Studies

**Figure 2:**
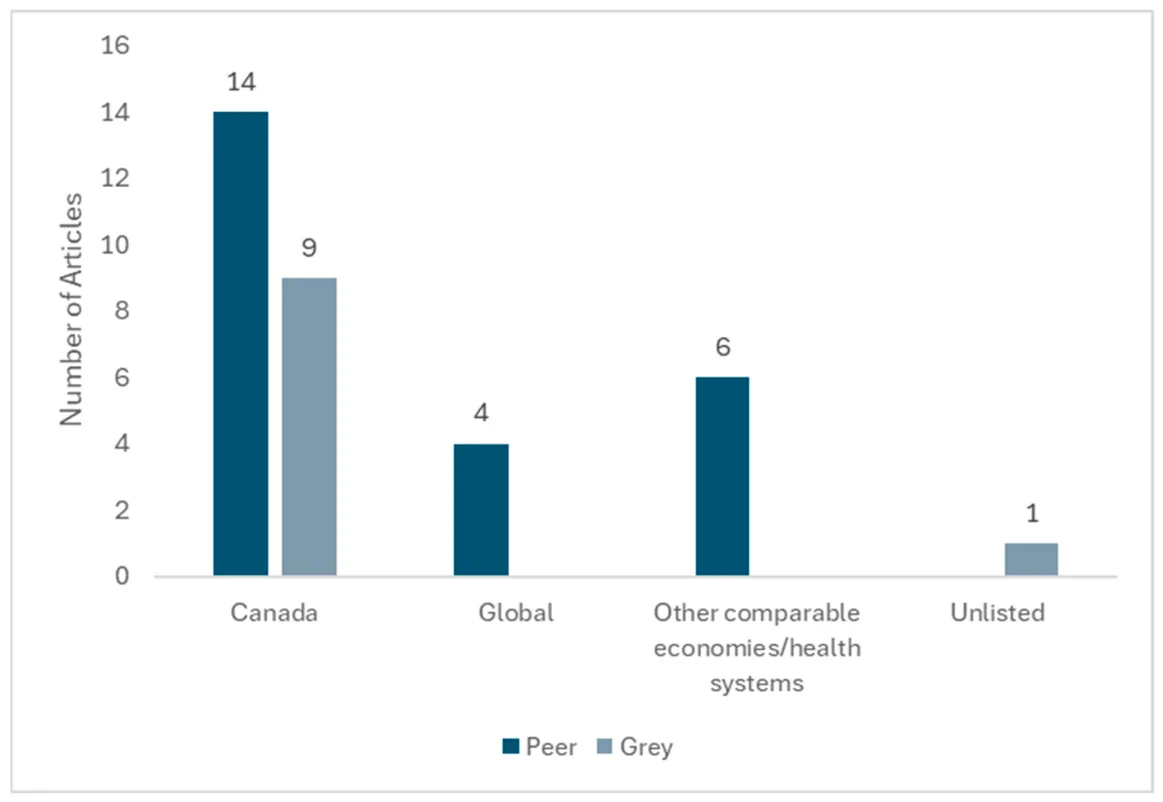
Geographic distribution of included studies (*n*= 34).

After the removal of duplicates, the search yielded 86 studies. Once screening commenced, 38 were excluded, leaving 48 studies screened for eligibility. After 24 articles were excluded, 24 peer-reviewed literature articles remained (Figure 1). An additional 10 grey literature articles were identified. Most studies for both peer review (*n* = 14/24, 58%) and grey literature (*n* = 9/10, 90%) were based in Canada. Other articles from outside of Canada were in the context of other comparable health systems or discussed multiple countries (Figure 2). In particular, they were from Iran, the Netherlands, New Zealand, France, China, and Indonesia (*n* = 6/24, 25%). A summary table of themes is available in Appendix B.

#### 3.1.1. Attachment and Influencing Factors

Attachment in primary care refers to a provider’s responsibility for continuous, comprehensive patient care, with the linking process known as enrollment, empanelment, or rostering [25,26]. Terminology varies globally, with China using the Family Doctor System with “contracting,” the Netherlands using “registration,” some countries using gatekeeping models, and others relying on voluntary attachment [27,28]. Strong primary care access supports equitable, cost-effective outcomes through prevention and early treatment [29]. Attachment is influenced by factors such as patient awareness, trust in the healthcare system, life transitions, provider attitudes, and cultural preferences, including mistrust of general practitioners compared to specialists [10,30,31].

#### 3.1.2. Barriers and Facilitators to Attachment

Attachment to primary care is shaped by a range of barriers and facilitators, particularly those related to the social determinants of health [32]. Factors such as immigration status, experiences of discrimination by service providers, and broader social and economic conditions can limit individuals’ ability or willingness to attach to a provider [33]. At the same time, membership in supportive communities can facilitate attachment by providing information, encouragement, and navigation support. Additional challenges, including a lack of knowledge about how to access primary care and logistical issues such as transportation, scheduling, and system complexity, further influence whether individuals can establish and maintain attachment [32,34].

#### 3.1.3. Characteristics of an Attachment-Ready Provider

An attachment-ready primary healthcare provider is one who has the capacity, through appropriate panel size, staffing, and team composition, needed to take on new patients while ensuring availability, longitudinal care, and coordination across the patient’s healthcare journey [35]. Their team and infrastructure support continuity through tools such as EMR tracking, follow-up systems, proactive outreach, and care coordination. They also have efficient onboarding processes that enable understanding patients’ histories and contexts, insurance verification, timely scheduling, patient education, and clear communication of clinic policies [35]. Beyond operational readiness, these providers demonstrate a commitment to meeting diverse patient needs, including a willingness to accept patients regardless of background or disease complexity, even though they tend to prefer less complex cases [8]. They take accountability for ongoing, continuous care, are comfortable working within patient-panel and team-based care models, and are equipped to address social and clinical complexity. Additionally, they support improved access by offering care beyond regular office hours and aligning with remuneration models, such as capitation, that account for patient size and complexity [26].

### 3.2. Findings from Qualitative Interviews

The scoping review findings were reinforced through a qualitative approach, using semi-structured, in-depth interviews to explore attachment readiness to primary care from the perspectives of patients and primary healthcare providers.

#### 3.2.1. Attachment Readiness from Patient, Provider, and System Perspectives

Attachment readiness emerged across all interviews as a process rather than a single event, shaped by interactions among patient need, provider capacity, onboarding practices, and system-level supports. Findings from patients, physicians, onboarding staff, and program managers collectively illustrate how readiness is constructed, constrained, and supported at multiple points along the attachment pathway.

#### 3.2.2. Patient Perspectives: Readiness Driven by Need, Experience, and Uncertainty

From the patient perspective, attachment readiness was rooted in the recognition that episodic or fragmented care was insufficient to meet ongoing health needs. Patients described specific triggers that prompted them to seek attachment, including the need for routine cancer screening, persistent unmanaged pain, hypertension, mental health concerns, and delays in diagnostic follow-up. Prior reliance on walk-in clinics was commonly described as inadequate for continuity, reinforcing the need for a consistent primary care provider.

Social and contextual experiences further shaped readiness. Patients described traumatic community events, legal and psychosocial challenges, and the COVID-19 pandemic as moments that clarified the importance of having an attached provider capable of understanding complex, intersecting needs. For these patients, readiness was not abstract but urgent and grounded in lived experience.

Despite both patients’ and providers’ readiness, patients described system-level barriers that disrupted attachment. These included long waits for acceptance, delays in scheduling a first appointment, and repeated requests to restate information already provided in shared records, all of which extended onboarding time.

While many patients felt personally prepared to complete registration and intake forms, their readiness was undermined by prolonged waiting periods between application and acceptance, as well as other barriers, including language and digital literacy (Table A1 in Appendix A). These barriers persisted for individuals when completing intake and registration forms and booking appointments online. The reported wait times ranged from one to seven months, during which patients experienced uncertainty, limited communication, and difficulty identifying whom to contact for updates. Inconsistent information from front-desk staff further contributed to frustration and, in some cases, regret over changing providers. These delays and ambiguities strained readiness despite patients’ motivation to attach.

Patients also identified communication and system navigation as ongoing challenges even after attachment. Difficulty reaching clinics by phone, unanswered calls, long hold times, and confusion about follow-up care disrupted continuity of care. In some cases, patients were removed from clinic rosters after a prolonged absence, without a clear understanding of their status, resulting in re-registration and further destabilizing attachment.

Despite these barriers, patients reported that attachment readiness was strengthened when onboarding staff and providers demonstrated empathy, patience, and preparedness. Reviewing medical histories in advance, facilitating interpreter support, allowing sufficient time during first visits, and addressing both physical and psychosocial concerns reinforced patients’ sense of being understood and supported. The first clinical encounter proved critical in solidifying readiness and attachment. Patients also highlighted the need for greater onboarding capacity, dedicated staff to manage intake and phone communication, and prioritization based on urgency or clinical need.

#### 3.2.3. Physician Perspectives: Readiness Constrained by Capacity and Administrative Burden

Physicians described attachment readiness as closely tied to clinic capacity, administrative workload, and the availability of support structures. In private community clinics, onboarding new patients was characterized as substantial, unpaid and often invisible work, encompassing chart creation, consent management, portal registration, medication reconciliation, and retrieval of external records. This work competed directly with clinical responsibilities and influenced decisions about accepting new patients.

Physicians emphasized deliberate efforts to structure onboarding through meet-and-greet appointments, policy explanations, and repeated communication of expectations. However, reliance on voicemail and email disadvantaged patients with limited digital access, necessitating repeated explanations and increasing workload. Capacity limitations led many clinics to restrict acceptance to specific groups, such as children or family members of existing patients, highlighting ethical tensions between the desire to help and practical constraints.

In community health centres and team-based models, readiness was shaped differently but remained constrained by system factors. Physicians reported limited real-time information on onboarding timelines, eligibility criteria, and capacity changes, which reduced their ability to provide clear guidance to patients. Staffing changes, such as leaves, could rapidly alter onboarding capacity, creating pauses that were difficult to communicate transparently.

Across settings, physicians identified administrative overload as a persistent barrier. Even when supportive intake roles were introduced, workload shifted rather than disappeared, often without additional staffing. Bottlenecks during first visits—including insufficient buffer time, late arrivals, and missing records—further strained readiness and disrupted clinic flow. Physicians consistently noted that attachment readiness would improve when information was gathered, organized, and entered into electronic medical records prior to the physician encounter.

From the onboarding staff’s perspective, attachment readiness began when patients recognized the need for a family doctor and submitted an application. Readiness was conceptualized as a process dependent on accurate assessment, eligibility screening, and completeness of information, rather than as a single step. Onboarding staff emphasized prioritization and fast-tracking those needing immediate care, while processing standard intake pathways. Otherwise, there may be a risk of “readiness decay” among patients, particularly if they are newcomers, refugees, or members of equity-deserving populations.

Staff reported the need to repeat and rephrase questions to verify accuracy, particularly when language barriers, literacy challenges, or interpreter-mediated communication were present. Even English-speaking patients sometimes provided excessive or irrelevant documentation, increasing intake time. In cases involving illiteracy, staff relied on family members or children, further extending onboarding appointments.

Onboarding staff acknowledged that repeating questions already captured on registration forms was necessary to ensure reliability for physician screening. Obtaining prior medical records with patient consent added further coordination demands. While most patients were described as cooperative, inconsistent or unclear responses delayed progression through onboarding and affected readiness.

Onboarding staff suggested that earlier physician involvement and more structured registration forms could improve efficiency and reduce task repetition. However, they clarified that intake remained primarily administrative, with physicians assuming responsibility for detailed clinical questioning later in the attachment process.

#### 3.2.4. Program Manager Perspectives: Readiness as a System-Enabled Condition

Program managers framed attachment readiness as a system-supported, relational process contingent on the alignment of patient preparedness, provider capacity, and intermediary supports. In private clinic contexts, readiness was enhanced through attachment liaison roles, often staffed by registered nurses, who conducted early assessments, consolidated histories, completed preventive care tasks, and coordinated referrals prior to the first physician visit. These supports reduced physician burden and increased willingness to accept new patients.

Within community health centre settings, readiness was described as emerging through holistic engagement and stabilization rather than discrete onboarding events. Roles such as holistic navigation counsellors supported patients with intersecting health and social needs, fostering continuity and trust before formal attachment. In this model, readiness is developed through sustained engagement rather than immediate physician attachment.

Across both contexts, program managers emphasized the importance of intermediary roles, centralized intake mechanisms, standardized data collection, and structured communication. Readiness was consistently described not as an individual attribute but as an emergent outcome of coordinated systems that supported both patients and providers at the point of attachment.

### 3.3. Triangulated Results

Below is a synthesis of the attachment efforts across Canada pilots and international models found in our scoping review and interviews (Table 1). Findings were consolidated separately to identify key characteristics including facilitators and barriers related to primary care attachment. These findings were then converged to develop and refine core themes. Interview findings further provided contextual insight that extended patterns identified in the literature. A full summary of these models is available in Appendix A.

Conversely, the literature identifies facilitators that enhance readiness, including team-based primary care models, patient navigation supports, culturally responsive outreach, and structured onboarding processes. Collectively, these findings support the conceptualization of attachment readiness as a system-enabled condition that must be intentionally supported through coordinated policy, organizational design, and practice-level interventions.

Our qualitative findings support these findings. Even when patients are motivated, informed, and willing to attach, prolonged waiting periods and lack of communication undermine their readiness. This aligns with evidence that unattached patients experience frustration, disengagement, and a breakdown in trust during prolonged attachment processes. Attachment is not solely constrained by physician supply but is also limited by readiness conditions that shape whether attachment efforts succeed or fail. Our interviews also highlighted readiness as time-sensitive, suggesting that attachment initiatives that do not account for delays risk losing prepared patients, a dimension less explicitly addressed in prior work.

## 4. Discussion

This study and analysis were conducted using data on residents of Ontario; however, they have significant implications for equity-deserving populations, such as newcomers, refugees, and people without status. Across jurisdictions and health system models, attachment readiness emerges as a relational, process-oriented, and context-dependent construct. While attachment is widely defined as the formal enrollment or registration of patients with a primary care provider or team, the literature consistently indicates that attachment alone does not ensure effective or sustained primary care relationships. We tested the strategies against the context to move Canadian primary care policy from a “volume-based matching” model to an “equity-driven readiness” model that ensures sustainable longitudinal patient–provider relationships. Current centralized waiting lists (CWLs) often treat attachment as a binary administrative event (matched/unmatched). However, evidence shows that without readiness, these matches frequently fail, leading to:

- Patient Disengagement: Trust erodes during long wait times with zero communication.
- Provider Burnout: Excessive uncompensated administrative labour during onboarding.
- System Inequity: Marginalized populations face higher barriers to establishing stable care.

The review further demonstrates that readiness is shaped by the interaction of patient-level factors (such as health literacy, trust, documentation, and logistical capacity), provider-level factors (including panel capacity, staffing, onboarding processes, and willingness to accept complex cases), and system-level conditions (such as workforce supply, centralized waiting-list design, and availability of team-based supports). Our interview findings corroborated the scoping review findings that patient readiness is influenced by health literacy, trust, documentation, and logistical capacity. These interrelated factors indicate that readiness cannot be reduced to a singular administrative act such as registration or waitlist placement.

The review further highlights that attachment initiatives focused primarily on increasing capacity or accelerating patient–provider matching often overlook readiness-related barriers that undermine sustainability. Structural challenges—including workforce shortages, administrative fragmentation, inequities linked to social determinants of health, and inconsistent onboarding practices—continue to limit the effectiveness of attachment efforts with equity needs.

Aligning with the existing literature, our findings underscore that policies aimed solely at increasing attachment numbers risk overlooking the foundational role of readiness. Integrating readiness assessments into centralized waitlist algorithms, standardizing electronic onboarding, and supporting providers with capacity-building resources are recommended to improve attachment rates, reduce wasteful disengagement, and advance equitable continuous primary care.

**Figure 3:**
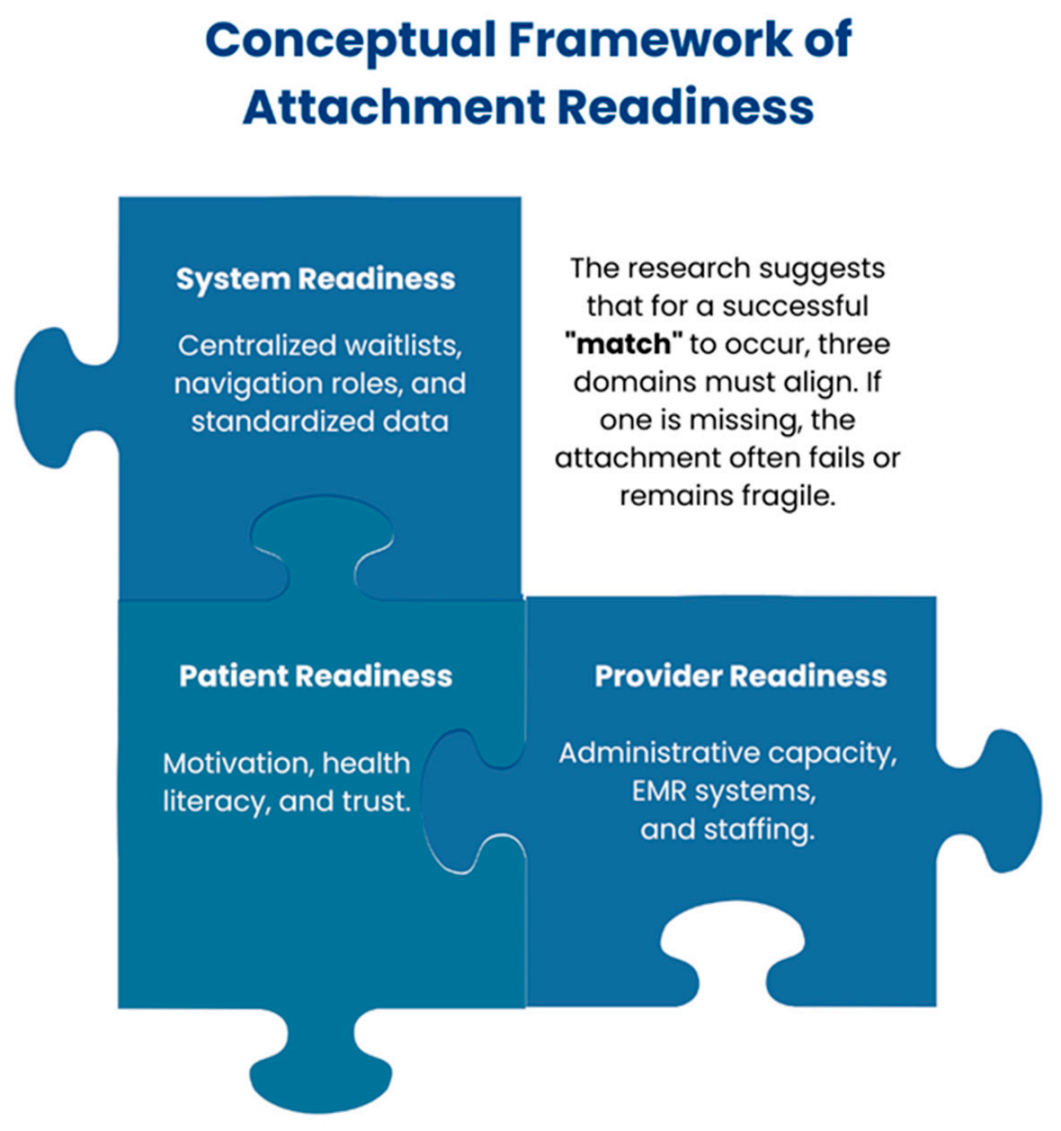
Conceptual framework of attachment readiness.

Findings across patients, providers, onboarding staff, and program managers demonstrate that attachment readiness is a multi-level, process-oriented construct shaped by information flow, capacity, communication, and system design (Figure 3). Attachment is successful only when the three domains of readiness intersect simultaneously. At the patient level, the strategic priority is to enhance health literacy and build trust in the system through transparent communication. At the provider level, efforts need to focus on strengthening clinic infrastructure and reducing administrative burden, particularly through EMR optimization. At the system level, integrating intermediary “navigator” roles is essential to bridge the gap between patient registration and the first clinical encounter (Figure 3). While patients often arrive motivated and prepared, readiness is constrained by waiting periods, administrative complexity, and unclear communication. Providers and staff navigate competing demands and limited resources, while system-level supports play a critical role in enabling sustainable attachment. From all perspectives, attachment readiness is best understood as a shared condition arising from coordinated onboarding processes rather than from individual readiness alone.

After analyzing all data, this study finds that effective policy and implementation need to clearly distinguish attachment readiness from attachment itself, recognizing readiness as a distinct construct across patients, providers, and systems rather than assuming that enrollment alone signals preparedness. Current policy metrics often treat attachment as complete once a patient is formally linked to a provider’s billing number; however, this is a lagging indicator that overlooks whether patients are functionally prepared to sustain a longitudinal care relationship. Our findings suggest that attachment must be preceded by readiness, understood as the patient’s capacity, confidence, and contextual ability to engage in ongoing primary care. As such, health systems should adopt Readiness Key Performance Indicators (KPIs) as leading indicators of successful attachment, including measures such as time from registration to intake completion and patient preparedness scores derived from pre-appointment surveys. Tracking readiness in this way would enable decision-makers to identify systemic bottlenecks, such as prolonged “in-between” stages, before these delays lead to failed or unstable attachments, particularly given that readiness is time-sensitive and may decay amid prolonged uncertainty during onboarding.

To operationalize this shift (Table 2), readiness assessments should be embedded into centralized attachment mechanisms such as waiting lists and registries, with standardized data collected at intake and updated over time to improve prioritization and reduce mismatches. At the system level, investment in infrastructure is essential to support readiness across all actors, including interoperable data systems, adequate staffing, and sustained intermediary roles such as navigators and onboarding teams, with successful pilots scaled and replicated across similar organizations. Strengthening patient readiness further requires clear, multimodal communication and navigation support to ensure that information about processes, timelines, and requirements is accessible through both digital and non-digital channels. Provider and practice readiness should be reinforced through structured onboarding, team-based care models, reduced administrative burden, appropriate EMR access for interdisciplinary staff, and alignment of attachment targets with realistic capacity. Standardizing data collection, onboarding workflows, and feedback loops across initiatives enhances consistency and transparency, while ongoing research should continue to refine readiness concepts and tools and embed these measures into evaluations to better understand what drives success or failure.

### 4.1. Role of Digitalization and Scope of Using AI

The above-mentioned options of a centralized intake and a culturally responsive attachment system can be ensured through credible digitalization and an AI platform. Currently, healthcare providers at different levels, such as Family Teams, Community Health Centres, and regional hospitals, use non-transferable EMRs (such as PSSuite) and digital communication or registration platforms (such as OceanMD). Data between these institutions are not transferable, causing clinical service disarray and an administrative burden when attaching new patients. Appropriate use of AI and transferable platforms for data collection and retention is required.

### 4.2. Limitations

The articles that were included were in English only and excluded other languages and definitions that may have been relevant to attachment and primary care systems. Further methodological limitations include varied timeframes, geographic distribution, and reviewers. Timeframe limitations may have excluded relevant policy documents, although an approach prioritizing current literature was taken. Most of the included studies were conducted in Canada, therefore limiting the generalizability of these findings to other contexts and health systems. Further, the barriers and challenges to attachment identified in this review may not adequately reflect the experiences of underrepresented regions. While health systems mobilize to increase patient attachment to primary healthcare, there is a scarcity of studies on attachment readiness, which plays a vital role in successful patient attachment to primary healthcare providers. Moreover, as scoping reviews typically have two reviewers for screening and validation, some articles may not have been captured in this study, and inter-rater reliability was not present. Limitations also persisted in qualitative interviews, including limited geographic settings, which may reduce transferability to other jurisdictions. Given the small number of participants, not all views may be represented. Further, the proposed conceptual framework has not yet been prospectively validated in independent healthcare systems.

## 5. Conclusions

This study contributes to the growing body of literature on primary care attachment by empirically examining attachment readiness from the perspectives of patients, physicians, onboarding staff, and program managers. The implications are more for newcomers, refugees, and other equity-deserving populations. Consistent with existing research, the findings demonstrate that attachment readiness is not a single event or an individual attribute but a multilevel, process-oriented condition, e.g., patients, providers, and the system design.

Patients in this study were often motivated and willing to attend; however, readiness was undermined by prolonged waits, unclear communication, and administrative complexity. Providers expressed willingness to accept new patients but identified administrative burden, staffing constraints, and workflow pressures as key limits to readiness. At the system level, structured onboarding roles, centralized coordination, and team-based models emerged as critical facilitators of sustainable attachment, while fragmented processes and limited resources constrained effectiveness.

Aligning with the literature, these findings underscore that policies aimed solely at increasing attachment numbers risk overlooking the foundational role of readiness. Attachment initiatives that fail to address readiness may lead to disengagement, inefficient use of resources, and inequitable access, particularly for patients with complex needs.

This study reinforces the importance of reframing attachment as a system-enabled relationship rather than a simple administrative match. Embedding attachment readiness into policy design, onboarding processes, and primary care reform efforts is essential to advancing equitable, continuous, and sustainable primary healthcare.

In conclusion, achieving equitable, sustainable, and effective primary care in Canada depends on fostering an “attachment-ready ecosystem”. By addressing the barriers identified in this research—ranging from systemic (administrative) burdens on physicians to logistical hurdles in intake—healthcare systems can ensure that, when a patient’s and provider’s needs are each identified, acknowledged, addressed, and finally linked through formal attachment, they are both prepared to sustain a continuous, high-quality healing relationship. By addressing the barriers identified in this research—ranging from systemic physician shortages to the logistical hurdles of intake—healthcare systems can ensure that when a patient and provider are finally linked, they are both prepared to sustain a continuous, high-quality healing relationship.

## 6. Recommendations for Next Steps: Operational Level

To move beyond fragmented care and reliance on high-cost emergency departments, Canadian healthcare policy must evolve. Enhancing attachment requires:

1. Ongoing “Realist Evaluation” for Sustainability: This current, more political, “rapid attachment” strategy has been promulgated as a heuristic decision-making tool to support the ailing residents, conducive to improving the public health system, productivity, and reducing the high burden on the healthcare system. The implementation was rapid and unsynchronized, requiring multi-level, complex evaluation matrices to ensure sustainability. Access Alliance and Alliance for Healthier Communities can jointly conduct this multi-metric evaluation going forward.
2. Standardizing Onboarding: Implementing uniform digital intake processes to reduce repetitive data entry and administrative friction.
3. Prioritizing Vulnerability: Refining centralized waitlist algorithms to account for clinical urgency and social determinants of health rather than simple chronological order.
4. Supporting Provider Capacity: Providing financial and administrative incentives that recognize the labour involved in establishing new longitudinal relationships, particularly for patients with complex needs.

## 7. Recommendations for Next Steps: System Transformation Level

1. Funding for evaluation for sustainability with redefined metrics: Implement “Readiness Indicators” (e.g., patient health literacy scores, clinic onboarding capacity) as distinct KPIs from raw enrollment data. This shift in the paradigm can ensure a transition from a quantity-based to a value-based framework.
2. Fund for intermediary roles: Institutionalize Primary Care Attachment Liaisons and Holistic Navigators to manage the “in-between” stage of the patient journey.
3. Modified intake algorithm: Create a uniform, provincial digital onboarding protocol to reduce redundant data entry and unpaid physician labour.
4. Diligence to protect trust: Deploy automated, multimodal updates for patients on waiting lists to prevent “readiness decay” and loss of system confidence.

## 8. Recommendations for Next Steps: Future Validation Studies

- Future research should validate, as an implementation research agenda, the “Primary Care Attachment Readiness Scale for Newcomers and Equity-Deserving Residents” (PCARS-NER). The evaluation matrices can be:
  1. Success of attachment (e.g., #new clients, #longitudinal continuity, access utilization, etc.)
  2. Attachment readiness (e.g., awareness, administrative readiness, digital readiness, referral, follow-up, etc.)
  3. Readiness scoring model (e.g., high, medium, low, etc.)
  4. Policy-relevant system (e.g., %unattached, median wait time for attachment, %vulnerable attachment, 12-month sustainability, ER use among attached Vs. unattached, etc.).
- This follow-up research will be a longitudinal study with strong policy advocacy components.

## Figures and Tables

**Table 1:** Core themes identified, combining findings from scoping review and interviews.

Theme	How It Shows Up in the Examples	Why It Matters
Centralized intake/single-point registration	CWLs in PEI, Quebec, Ontario, NB, BC, Nova Scotia, Alberta, PAI; BC PAS Health Connect Registry; England/France “gatekeeper” enrolment	Reduces fragmentation, provides health system planners with a real-time view of demand, and makes the process transparent to patients.
Standardized assessment & prioritization	Quebec’s A-E algorithm; Ontario’s health questionnaire; Alberta PAI’s onboarding interview; BC RAP’s clinical-risk triage; Iran’s family physician program	Allows scarce provider capacity to be directed toward the most vulnerable or complex patients and creates an objective basis for matching.
Explicit matching algorithm or coordinator role	Ontario’s algorithmic scoring; Manitoba’s coordinator-driven fast match; BC PAS coordinators; concierge model nurse in Ontario pilot	Human or automated matching improves speed and fit (geography, gender preference, chronic disease expertise) compared with pure “first come first served”.
Financial or non-financial incentives for providers	Quebec’s incentives for vulnerable patients; NB’s requirement to attach 600 CWL patients; BC’s new-to-practice incentive; Alberta’s panel-capacity flex-funding	Incentives motivate providers to open slots for unattached patients and to take on more complex cases that they might otherwise decline.
Data integration with existing health system records	Quebec’s link to RAMQ insurance database; Ontario’s Healthcare Connect data feeds; BC’s Panel Registry linked to provider capacity; Canada-wide EMR access in concierge pilot	Accurate, up-to-date data reduces duplicate registrations, keeps patient status visible, and enables analytics for planning.
Public awareness & easy access channels	Online portals (PEI, Quebec, NB, Nova Scotia); phone/811 lines (Manitoba, NB, Nova Scotia); limited online options (BC)—a cautionary note	Simpler registration lowers barriers for vulnerable groups (e.g., the elderly and those with low digital literacy) and improves uptake.
Dedicated staff or “concierge” role	Manitoba & BC coordinators; Ontario concierge nurse pilot; Alberta PAI staff; BC PAS coordinators; Flemingdon Health Centre, Midwest-Toronto Family Practice Network	A human point of contact can resolve data errors, answer patient questions, and keep the pipeline moving when algorithms stall.
Flexibility for patient preferences	Alberta PAI (gender, location, practice type); BC PAS (regional & specialty needs); Ontario concierge (EMR set-up)	Respecting patient choice improves satisfaction and reduces drop-out after attachment.
Monitoring, reporting & sustainability planning	Quebec’s strong reporting tools; Ontario’s regional monitoring; Nova Scotia’s panel-identification phases; BC’s $692 M investment with performance metrics	Ongoing evaluation lets programs adapt, justify funding, and demonstrate impact.

**Table 2:** Implications and key takeaways at different levels.

Level of Intervention	Implications
Primary Healthcare Organization	Tap existing organizational assets (e.g., registration portals, provider rosters, linked databases) to identify integration opportunities. Develop a minimum viable centralized platform with a secure web form, phone line, and basic triage system to prioritize patients. Pilot a rapid access stream for high-need patients, with dedicated slots and measurement of attachment and retention outcomes. Use early data to continuously refine triage, incentives, and staffing to address bottlenecks and improve performance.
Toronto Regions	Strengthen system capacity by investing in dedicated coordinators with read-only EMR access to support coordination and verification. Implement a tiered incentive framework with baseline payments and additional rewards for managing complex patients and ensuring retention. Establish centralized, real-time reporting dashboards to track key metrics. Publicly share data to improve transparency, accountability, and system performance.
Global Regions	Use a hybrid matching approach that combines technology with human judgment to balance efficiency and nuance. Prioritize data integration with existing health system databases to improve accuracy and reduce administrative burden. Implement tiered incentive structures to better support complex patients and avoid unintended effects of volume-based targets. Enhance transparency through public dashboards and clear communication to build trust and encourage enrollment. Pilot the model on a small scale, refine based on results, and then scale toward a centralized, coordinated, and equitable attachment system.

**Figure A1:**
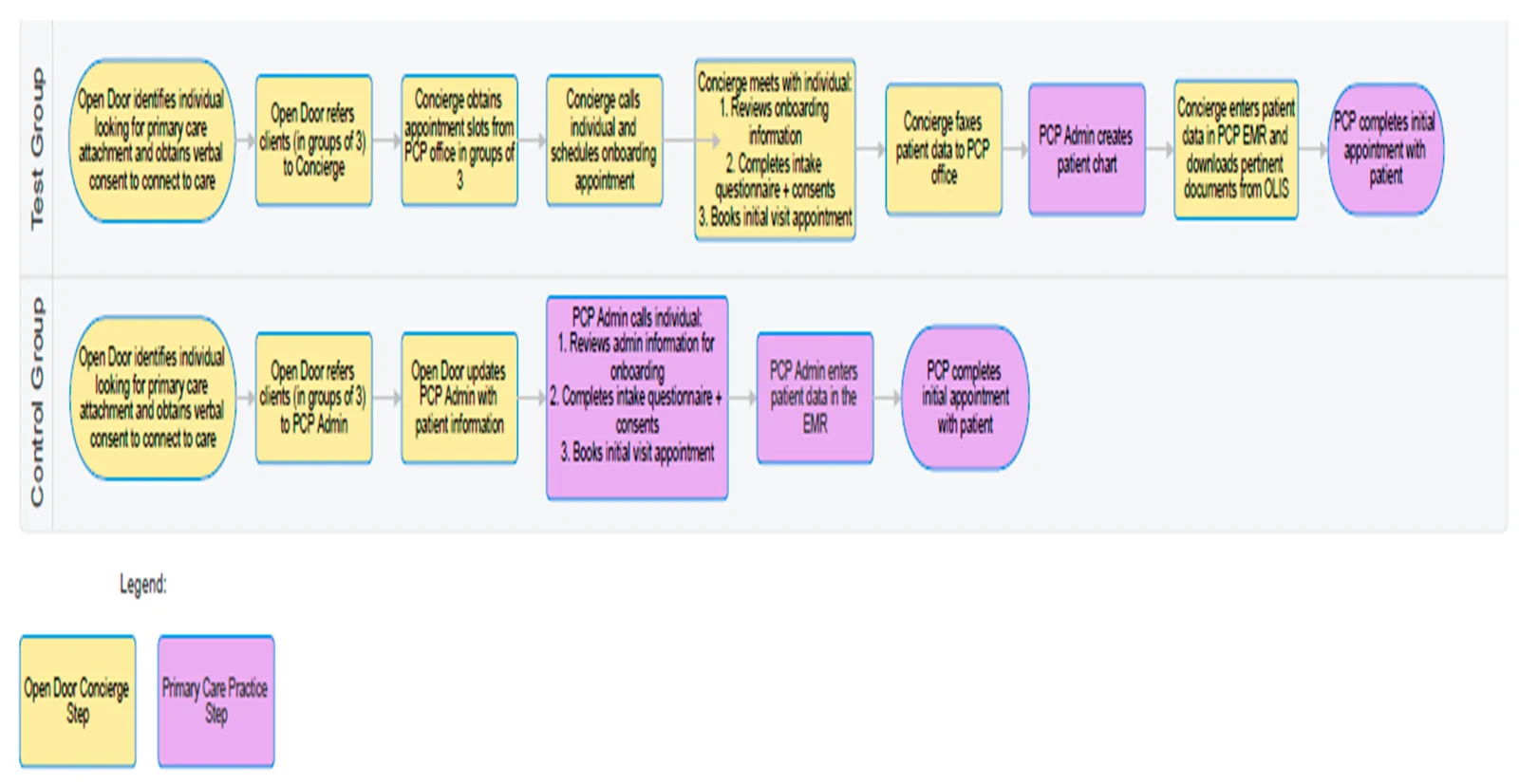
Concierge model versus the model without a concierge. Source: [36].

**Figure A2:**
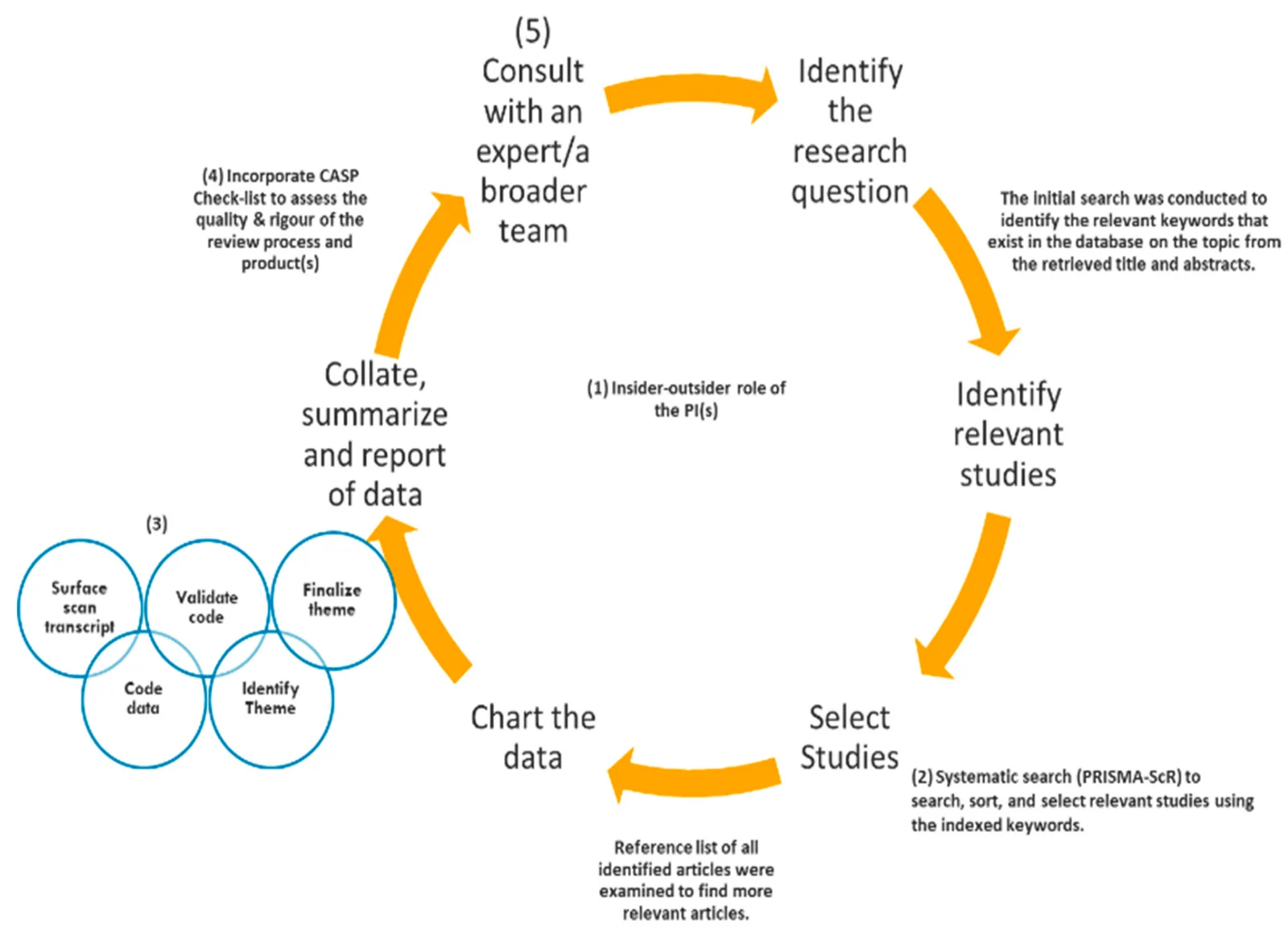
Scoping review model [18].

## Data Availability

Data are secured and stored in our encrypted repository, in accordance with the data confidentiality protocol. It is available and can be shared if requested.

## Appendix A. Existing Initiatives in Primary Healthcare Attachment

Millions of people remain unattached to primary healthcare, leading to fragmented care, delayed diagnoses, and unmet health needs. In response to this growing concern, governments, health authorities, and community organizations have developed a range of programs, policies, and pilot initiatives to improve attachment and strengthen continuity of care. These efforts are intended to mitigate the adverse consequences associated with being unattached and to enhance overall population health, equity, and system performance. Some prominent initiatives are discussed below:

### Appendix A.1. Centralized Waiting Lists

In a centralized waiting list (CWL), patients are registered at a single central intake point, then lined up and assigned to the next available service from a list of participating providers [8]. CWLs are seen as an important part of attaching patients to primary care to improve access.

**Table A1:** Centralized waiting lists in the seven provinces.

Province	Program Name	Implementation Year	Registration Process	Patient Assessment and Prioritization	Patient Attachment Process	Good Practice	Challenges
Prince Edward Island	Patient Registry Program	1998	Online + phone; simple system; reasonable duplication control	Basic prioritization for urgent cases/hospital discharges	Informal; physicians rewarded after panel exceeds 1200 patients	Easy registration, strong reporting tools, and high public awareness	Outdated provider lists; patient refusals; weak mechanisms for complex cases
Quebec	Guichets d’accès à un médecin de famille (Family doctor access point)	2008	Online or phone; linked to RAMQ insurance database	Automated priority levels A–E using self-report + RAMQ data; nurses review complex cases	Formal attachment to physician panels; financial incentives for attaching vulnerable patients	Best data integration; clear prioritization, strong incentives	Cherry-picking risk; difficult to attach complex patients; provider shortages; complicated detachment rules
Ontario	Healthcare Connect	2009	Online or phone; long process	Algorithmic scoring using a standardized health questionnaire	Formal attachment via Healthcare Connect; early incentives removed due to gaming	Highly standardized assessment; strong regional monitoring	Long waits, low public awareness, vulnerable patients struggle with forms, and provider shortages
Manitoba	Family Doctor Finder	2013	Phone registration with clerks; easy access	Limited prioritization (newborns, hospital discharge); health info cannot be updated later	Informal but fast matching using coordinator knowledge; high attachment success	Strong coordinator relationships; fast attachment (≈95% within 30 days)	Manual systems; outdated info; complex cases hard to attach; staffing shortages
New Brunswick	Patient Connect NB	2013	Online or 811 phone registration	No prioritization; first-come, first-served	Informal; new physicians required to attach 600 CWL patients; incentives provided	Very transparent; simple process; strong incentives for new doctors	Sick/vulnerable patients wait too long; high refusal rate; long wait times
British Columbia	A GP for Me, Health Connect Registry, RAP	2015	Phone registration; no online option; regional CWLs	No formal criteria; prioritization done subjectively by coordinators	Informal; strong local coordinator–provider matching	Local autonomy; tailored to community needs	Limited data (do not track waitlist size); temporary funding; inconsistent processes; hard to attach complex patients
Nova Scotia	Need a Family Practice	2016	Online + 811 phone; mainly tracks demand	Minimal prioritization; health changes are not easily updated	Informal; attachment not guaranteed; coordinators shift supply across regions	Strong monitoring; aligned with collaborative care teams	Limited staff; outdated provider info; very long waits; complex cases difficult to attach

The objective of these CWLs was to reduce the number of unattached patients by attaching them to a primary healthcare provider through a centralized channel. It was also a way of finding out who wants to be attached and, accordingly, measuring demand without the obligation to have them attached and ultimately making the healthcare system more accessible, continuous, and comprehensive (five principles of the healthcare system). CWLs are implemented in three stages:

- Registration of the patients.
- Assessment of the patients.
- Attachment to the matching provider.

Implementation of the CWLs encountered systemic challenges at each stage of registration, assessment, and attachment.

Limited staffing and high call volumes, outdated or manually managed systems, and low overall public awareness of waitlists made registration challenging. In many areas, registration was conducted by phone with no online options, resulting in long wait times and inaccurate information. Patients also frequently encounter difficulties with lengthy or complex forms, and many are unaware of their current attachment status. Inadequate follow-up and outdated patient or provider contact details further complicate registration efforts.

Assessment was hindered by the limited or overly simplistic prioritization methods, and in some cases, it was carried out entirely on a first-come, first-served basis. Even when assessments are in place, patients may misreport or exaggerate their symptoms to try to increase their priority. Staff often lack the authority to collect detailed health information or to update a patient’s priority status when their condition changes. Without standardized assessment tools, decisions become subjective and inconsistent, and because the process relies on manual data entry, errors and outdated information are common.

The attachment stage faced difficulties due to provider shortages, especially in rural or underserved areas, resulting in long waits and limited capacity to place patients. Providers frequently decline complex cases, including those involving mental health or chronic conditions, creating inequitable attachment outcomes. Inefficiencies arise from outdated or manually maintained provider availability lists, while short booking windows and the absence of tracking for informal attachments leave additional gaps in the process. Sustainability concerns, such as temporary funding and inadequate monitoring systems, further undermine long-term stability. As a result, complex patients encounter the most significant barriers, and the system continues to struggle with consistency, fairness, and timely placement.”

### Appendix A.2. Alberta Patient Attachment Initiative (PAI)

The Alberta Patient Attachment Initiative (PAI) helps attach patients with complex needs to a primary care provider (PCP) through referrals. PAI maintains panels for the participating PCP with flexibility in patient types and demographics.

The process involves:

1. Referral received: Referral of an unattached patient to PAI. PAI staff records the referral and confirms eligibility.
2. Assessment: An onboarding assessment is carried out through a phone call or an online form to determine the care needs and preferences of the patient, including the location of the PCP, gender, etc.
3. Match request: An anonymized request is sent to the most suitable PCP for the patient, ensuring it matches the PCP’s panel need and capacity.
4. Acceptance and Package: On acceptance by the PCP, a package is sent to the PCP’s place of practice with the referral letter, acceptance by the PCP and patients’ medical records. The patient is advised to book their first appointment within 4 weeks of referral.

Some of the factors that affected attachment were:

- Educating patients about attachments and the importance of notifying their PCP if they end the relationship, which could minimize conflicts between panels.
- A more structured and standardized panelling approach across a network of clinics or at the provincial level would help mitigate conflicts.
- Panelling regularly.
- Patient’s autonomy to continue or terminate the attachment.
- The PCP and his practice’s characteristics, the patient’s overall health condition or the general attitude of the patients regarding the requirement for the PCP.
- Gender of the PCP, as some patients prefer to consult a PCP of a specific gender due to the nature of their health issue.
- Patient not understanding the importance of attachment, although they should be given the flexibility to seek care from other PCPs
- The patients might have unmet mental needs, hence their seeking care from multiple PCPs.

### Appendix A.3. British Columbia Patient Attachment System (PAS)

This system is a coordinated approach by the provincial government, Doctors of British Columbia, and Nurses and Nurse Practitioners of BC to match patients with physicians who are accepting new patients. The process is further supported by recruiting additional coordinators who will try to match registered individuals and families with available providers locally.

This is carried out through two interfaces:

- Health Connect Registry: Interface for patients who are ready to be attached.
- Panel Registry: Interface for family physicians.

There is another interface for medical directors of clinics (team-based care) called the Clinic and Provider Registry.

The patients register through the Health Connect Registry, and concurrently, providers, especially new family doctors or nurse practitioners, register through Panel Registry. New clinics or team-based care providers register through the Clinic and Provider Registry. Patients provide information such as their geographic region and healthcare needs, while providers provide details of their practice, available patient capacity, and willingness to accept new patients. Providers detail their practice, available patient capacity, and willingness to accept new patients. Patients are matched with providers based on the patient’s healthcare needs, the provider’s ability to take on those needs, and the region.

The provincial government is taking significant action to expand this initiative throughout the province by strategies such as a new-to-practice incentive program for early-career doctors. The government is also investing over $692 million to expand team-based care, including the development of Primary Care Networks and Urgent and Primary Care Centres. Furthermore, the Health Human Resources Strategy is actively working to recruit, train, and retain healthcare workers, including making it easier for internationally educated professionals to work in BC, while empowering pharmacists to manage minor ailments to ease the burden on primary care.

There is another registry for patients who need specialized care: the Rapid Access for Patients (RAP) program. Patients on the RAP waitlist are often already receiving care from specialty healthcare services or require a primary care provider to coordinate care with specialty providers. RAP patients take priority over Health Connect Registry HCR patients when it comes to attachment and must be referred by a healthcare provider on a case-by-case basis. Priority is given to urgent and vulnerable patient populations, such as frail adults, new moms and infants, mental health and/or substance use patients, moderate-to-high needs individuals, and those with complex chronic conditions. The attachment wait time is approximately four months.

### Appendix A.4. Nova Scotia Panel Identification

Nova Scotia Health maintains a list of patients for whom primary healthcare providers are responsible. The main aim of this process is to maintain the relationship between the patient and the care provider to provide better primary care, preventive care, timely access to care, continuity of care, team-based care, and allow performance measurement.

This process of attachment and panel identification is carried out in three phases:

- Phase 1: Identify: Includes steps to identify an accurate, up-to-date list of patients on a provider’s panel.
- Phase 2: Adjust: Guides determining whether supply and demand are in balance, informs conversations about panel size adjustments, and supports.
- Phase 3: Support and sustain: Establishes mechanisms for ongoing maintenance of the panel to sustain improvements.

### Appendix A.5. Concierge Model Pilot Project [36]

The Concierge Pilot Project was a collaboration between the Mid-West Toronto Ontario Health Team, Access Alliance Multicultural Health and Community Services, MyFamilyMD, and the Open-Door Program (Figure A1). It evaluated the practicality and effectiveness of the concierge role in managing the onboarding process of new patients in a non-group primary care practice. A registered nurse carried out the concierge role.

The concierge collected patient histories, educating patients about the practice and setting up their electronic medical records (EMR).

The project divided participants into two groups: a test group onboarded through the concierge process, and a control group onboarded directly by primary care providers.

The Concierge Pilot Project examined the feasibility of using a registered nurse to support patient onboarding in a non-group primary care setting, assessing time efficiency, provider experience, and patient satisfaction. Led by Access Alliance Multicultural Health and Community Services in collaboration with MyFamilyMD under the Mid-West Toronto Ontario Health Team, the pilot involved twelve patients divided into two groups: six patients supported by the concierge and six onboarded directly by primary care providers. Providers noted that having patient information collected in advance improved efficiency and quality of care, and patients reported high satisfaction with the clarity and friendliness of the concierge-supported onboarding experience.

Overall, the model was reported to improve care providers’ workflow, be time-efficient, and increase patients’ satisfaction. There were a few challenges due to logistical constraints and differences in data organization preferences among the providers. Some recommendations that came out of this project are:

- The concierge staff person should have remote access to the primary care providers’ electronic medical record (EMR).
- Having access to Ontario’s centralized waiting list would be useful.
- The concierge nurse role should be dedicated and focused on helping manage administrative tasks.

### Appendix A.6. International Attachment Examples

Different names across countries refer to the same concept: attaching patients to primary healthcare.

In England, France, the Netherlands, and Indonesia, a “gatekeeper” model exists, in which a general practitioner must be chosen as the primary doctor [26,27]. Without a referral from the gatekeeper, it is difficult for patients to access specialist (secondary and tertiary) healthcare.

In China, a Family Doctor Contracting System (FDCS) exists, where registration is called contracting. To increase the sign-up rate for family doctors, a tailored approach has been implemented that addresses the varying needs of different communities, using financial incentives such as higher insurance reimbursements, lower out-of-pocket payments, and loyalty discounts for repeat patients to mitigate individual concerns.

The Family Physician Programme and Referral system in Iran was initiated in remote areas and later rolled out in urban areas to provide effective, preventive, and curative care with fewer resources. This program was a strategy to achieve the World Health Organization’s (WHO) goal of “health for all” and involved establishing a healthcare insurance system [31].

In Israel, patients are free to choose from four private nonprofit health plans. Patient registration is limited to Chalit Health Plan, the largest of the four health plans. Patients with this health plan are automatically registered to a local clinic in their district. This plan adopts the gatekeeper system to emphasize the importance of continuity in primary healthcare [26].

In Sweden and Italy, patients are attached to a PCP. In Italy, patients have the freedom to change PCPs. In Denmark, it is voluntary to register with a PCP. However, most patients register to avoid the co-payment fee that all non-registrants are mandated to pay.

Ireland has an attachment system only for the poor and the elderly to provide free care via a medical card scheme, which requires patients to enrol with a GP practice.

## Appendix B. Literature Review Methodology

We used the scoping methodology and steps to carry out the review, as illustrated in Figure A2.

Databases and geographical regions to search for relevant papers are shown in Table A2 below:

**Table A2:** List of databases used for search.

Databases	Geographical Areas of the Primary Research
PubMed Search Premier MEDLINE (Ovid) ProQuest Science Direct JSTOR	Canadian Provinces Global (US, UK, Australia, other countries of Europe, China, Japan, and Iran)

**Search Method**

We used a Seneca College librarian’s help to ensure we followed appropriate search methods and found relevant papers, using the strategies outlined below.

Keywords for search: attachment, attachment readiness, primary healthcare, family doctor, nurse practitioner, team-based healthcare, community health centers/centres, MD, NP.

The filtered and selected data were then entered into the online database (MyBib.com) for managing reference articles and papers.

Inclusion Criterion:

- Articles relevant to attachment to primary healthcare providers.
- Year: 2010 to 2025, except for relevant articles or seminal articles.

Exclusion Criterion:

- Exclude articles not in English or with no English version available.
- Exclude tools/practices that are not grounded in evidence.

Quality check of the review process

We consulted the academic librarian for advice on adhering to the quality search and record system.

- The principal investigator (A.A.) played an insider–outsider role [20] in the review process.
- We adopted the PRISMA-ScR [18] flowchart to record and report the data search process.

**Summary of Findings**

**Supporting Article**	**Theme**	**Findings**
[25,26,27,28,29,30]	Attachment	- Attachment in primary care is a provider’s responsibility for a patient’s continuous and comprehensive care. - The process of linking patients with a provider is also known as enrollment, empanelment, or rostering. - In China: o The concept of attachment is called the Family Doctor System. o The process of attaching is called contracting. o And the relationship is called a contract. - In the Netherlands, the process is called registration. - The gatekeeper system in England, France, and Indonesia. - Voluntary attachment in the USA, Japan and Belgium.
[10]	Impacts of attachment	- Access to primary care is vital for fostering inclusive, equitable, and cost-effective health outcomes. - Provides preventive services, early detection and treatment of diseases.
[10,27,31,37]	Factors influencing attachment	- Patients’ awareness of the importance of primary care attachment. - Patient’s trust in the health system and institution. - Life transition, such as when moving from one province to another. - Mentality of the healthcare provider. - Culture of using services provided by family physicians and their mistrust of general practitioners in comparison to specialists.
[31,32,33,34]	Challenges/barriers and facilitators to attachment	- Social determinants of health. - Discrimination by service providers. - Immigration status. - Membership in communities. - Lack of knowledge. - Logistical issues.
[26,38,39]	Attachment Readiness	- For patients: - Willing to trust a specific provider. - Ready to attach to any doctor. - Willing to go beyond their geographic neighbourhood. - Are eligible to be covered by the National Health Service.
[8,35]	Characteristics of an attachment-ready PHC provider	- Efficient onboarding system. - Willing to take on patients regardless of their background. - Willing to be accountable for the patient’s longitudinal and continued care. - They are willing to extend access to the patients beyond regular office hours. - Willing to provide a basis for capitation-based remuneration.
[8]	Centralized Waiting Lists	Central intake point, then lined up and assigned to the next available service from a pool of participating providers.
[40]	Alberta Patient Attachment Initiative (PAI)	- Helps in attaching unattached patients with complex needs to find a PCP through referrals. - Attach each patient within a Primary Care Network’s (PCN) catchment area with a single Primary Care Provider.
[41,42]	British Columbia Patient Attachment System (PAS)	- Health Connect Registry: Interface for patients who are ready to be attached. - Panel Registry: Interface for family physicians providing longitudinal care. - Clinic and Provider Registry: Registry for the medical directors of clinics (team-based care). - Rapid Access for Patients (RAP) program.
[43]	Nova Scotia Panel identification	Maintain the relationship between the patient and the care provider to provide better primary care, preventive care, timely access to care, continuity of care, team-based care, and allow performance measurement.
[36]	Concierge Model Pilot	Integrating the role of a concierge, a practical nurse, to manage the patient onboarding process efficiently.
[27,31]	International Forms of Attachments	Gatekeeper model in England, France, the Netherlands, Israel and Indonesia. Family Doctor Contracting System in China. Family Physician Programme and referral system in Iran. Mandatory attachment, but freedom to change PCP in Sweden and Italy. Voluntary attachment in Denmark.
